# Structural Basis of the Induced-Fit Mechanism of 1,4-Dihydroxy-2-Naphthoyl Coenzyme A Synthase from the Crotonase Fold Superfamily

**DOI:** 10.1371/journal.pone.0063095

**Published:** 2013-04-26

**Authors:** Yueru Sun, Haigang Song, Jie Li, Yan Li, Ming Jiang, Jiahai Zhou, Zhihong Guo

**Affiliations:** 1 Department of Chemistry and State Key Laboratory of Molecular Neuroscience, The Hong Kong University of Science and Technology, Clear Water Bay, Kowloon, Hong Kong SAR, China; 2 State Key Laboratory of Bio-organic and Natural Products Chemistry, Shanghai Institute of Organic Chemistry, Chinese Academy of Sciences, Shanghai, China; Consejo Superior de Investigaciones Cientificas, Spain

## Abstract

1, 4-Dihydroxy-2-naphthoyl coenzyme A (DHNA-CoA) synthase is a typical crotonase fold enzyme with an implicated role of conformational changes in catalysis. We have identified these conformational changes by determining the structures of its *Escherichia coli* and *Synechocystis* sp. PCC6803 orthologues in complex with a product analog. The structural changes include the folding of an active-site loop into a β-hairpin and significant reorientation of a helix at the carboxy terminus. Interestingly, a new interface is formed between the ordered loop and the reoriented helix, both of which also form additional interactions with the coenzyme A moiety of the ligand. Site-directed mutation of the amino acid residues involved in these ligand-induced interactions significantly diminishes the enzyme activity. These results suggest a catalytically essential induced-fit that is likely initiated by the enzyme-ligand interactions at the active site.

## Introduction

**Figure 1:**
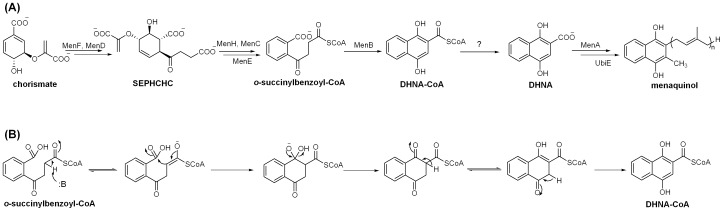
DHNA-CoA synthase (MenB) in the menaquinone biosynthesis. (A) The bacterial biosynthetic pathway of menaquinone (vitamin K2). SEPHCHC: (1*R*, 2*S*, 5*S*, 6*S*)-2-succinyl-5-enolpyruvyl-6- hydroxy-3-cyclohexene-1-carboxylate; OSB: *o*-succinyl-1-benzoate; DHNA: 1, 4-dihydroxy-2-naphthanoate; CoA: coenzyme A. (B) Intermediates in the proposed catalytic mechanism for DHNA-CoA synthase.

1, 4-Dihydroxy-2-naphthoyl-CoA (DHNA-CoA) synthase, also called MenB, is responsible for conversion of *o*-succinylbenzoyl-CoA to DHNA-CoA in the biosynthesis of both vitamin K1 and K2 [1], [2] (Figure 1A). It catalyzes a multiple-step intramolecular Claisen condensation reaction involving two high energy oxyanion intermediates and two keto-enol tautomerizations in the formation of a naphthenoid ring (Figure 1B). This complex chemical process is essential for many Gram-positive bacteria that rely on vitamin K2, or menaquinone, for electron transportation in the respiratory chain [1]. Disruption or knockout of the *menB* gene is lethal to important microbial pathogens such as *Haemophilus influenzae* and *Staphylococcus aureus* [3], [4]. Due to the absence of DHNA-CoA synthase in mammals, this enzyme has become an attractive target for the development of new antibiotics [5], [6], similar to other essential enzymes of the same biosynthetic pathway [7]–[10].

Recent investigations have revealed several characteristic structural and catalytic features of the vitamin K biosynthetic enzyme. The first crystal structure of DHNA-CoA synthase from *Mycobacterium tuberculosis* shows that it forms two eclipsed trimers organized in a homologous hexameric assembly, in which each monomer is comprised of an *N*-terminal spiral core domain and a C-terminal helical domain conforming to a canonical crotonase fold [11]. Interestingly, the C-terminal helical domain was found to cross the trimer-trimer interface to form part of the active site of the subunit in the opposite trimer. The same arrangement of the C-terminal helix is found in all the structurally known MenB enzymes, including those from *Staphylococcus aureus* [12], *Salmonella typhimurium* [13], *Geobacillus kaustophilus* HTA 426 [14], *Escherichia coli* [15], [16], and *Synechocystis* sp. PCC6803 [16]. This distinctive structural feature unites the MenB orthologues into a unique group within the crotonase superfamily. In other crotonase proteins, the C-terminal helical domain either folds back to the core domain of the same subunit or covers the active site of a neighboring subunit in a dimer or a trimer in the quaternary assembly [17].

Besides their structural uniqueness, DHNA-CoA synthases also show distinctive dependence on an exogenous anion in their catalysis. The activity of a large set of MenB orthologues, called type I enzymes, were found to completely depend on bicarbonate [18], [19], which has been found to occupy an equivalent position of the catalytic bases of other crotonase fold proteins and proposed to serve as a coenzyme to abstract an α-proton from the substrate to initiate the intramolecular Claisen condensation [13], [16]. The remaining MenB proteins are classified as type II enzymes, which show no response to activation of exogenous bicarbonate. In these MenB orthologues, the side-chain carboxylate of a conserved aspartate occupies the equivalent position of the exogenous bicarbonate in the type I enzymes and carries out the essential α-proton abstraction. No other crotonase fold protein has been found to use bicarbonate as a coenzyme like the type I MenB enzymes. Currently, bicarbonate is known to affect the activity of a limited set of enzyme systems such as cyclopropane fatty acid synthase [20], [21], photosystem II [22], amine oxidase [23], aminopeptidase [24] and adenylyl and guanylyl cyclases [25], [26].

Another interesting catalytic feature of DHNA-CoA synthases is the likely involvement of an induced fit catalytic mechanism suggested by previous structural and biochemical studies. Early on, it was noted that folding of a disordered active site loop found in the MenB crystal structure will seal the bound substrate off from the bulk solvent to protect the reaction intermediates, suggesting that the substrate may be able to induce conformational change of the protein [11]. Recently, two conserved residues of the disordered loop were shown to interact with bound product analog inhibitors through spectroscopic studies, implicating that the loop must be ordered when the inhibitors bind to the enzyme active site [27]. More recently, the crystal structure of the MenB enzyme from *Escherichia coli* in complex with the substrate analog *o*-succinylbenzoyl-amino coenzyme A (OSB-NCoA) was determined, which clearly shows that the active site loop is folded into a β-turn and a β-hairpin by the ligand [15]. In connection to this likely induced-fit catalytic mechanism for the DHNA-CoA synthases, it is interesting to note that a disordered active site loop is present at a similar position in rat mitochondrial crotonase [28], [29]. However, it is not known whether a similar induced fit underlies the catalysis of other members of the crotonase superfamily.

Despite the finding of ligand-induced conformational changes, it is not clear how the small molecule ligands induce these structural changes. To identify the crucial elements in the protein-ligand interactions, we determined the structure of the complexes of the enzymes from *Escherichia coli* and *Synechocystis* sp. PCC6803 with product analog 1-hydroxy-2-naphthoyl-CoA (HNA-CoA) or salicyloyl-CoA (SA-CoA). In addition to observing the folding of the active-site loop into a well-defined structure as previously seen in the substrate analog complex structure [15], we have identified a significant reorientation of the C-terminal helix as an additional conformational change caused by the small molecule ligands. Interestingly, the two altered protein moieties strongly interact with each other and both make additional contacts with the ligands. Through site-directed mutagenesis, we have collected evidence that the amino acid residues involved in these ligand-induced interactions are crucial to the enzyme activity, supporting a unique induced-fit catalytic mechanism for the DHNA-CoA synthases which involves intersubunit interactions.

## Materials and Methods

### Chemicals

The following reagents were purchased from Sigma: NaHCO_3_, 1-hydroxy-2-naphthoic acid, *N*-hydroxysuccinimide (NHS), *N*, *N*-dicyclohexylcarbodiimide (DCC), α-ketoglutarate, thiamine diphosphate, coenzyme A, adenosine triphosphate (ATP), isopropyl β-D-thiogalactopyranoside (IPTG), polyethylene glycol (PEG, average MW  = ∼3,350), 2-methyl-2, 4-pentadiol (MPD), buffers, and other salts. Chorismic acid was prepared using an engineered bacterial strain as described previously [30]. It was used to chemoenzymatically synthesize (1*R*, 6*R*)-2-succinyl-6-hydroxy-2,4-cyclohexadiene-1-carboxylate (SHCHC) with purified EntC [31], [32], MenD [33], MenC [34] and MenH [35] as described previously [36]. HNA-CoA and SA-CoA were synthesized respectively from 1-hydroxy-2-naphthoic acid and salicylic acid by a two-step coupling reaction [37].

### Protein expression and purification

The recombinant wild-type MenB enzymes from *E*. *coli* (*ec*MenB) and *Synechocystis* sp. PCC 6803 (*sc*MenB) were expressed and purified to homogeneity as previously described [18], [19]. The point mutants K89A, R267A, F270A, and K273A of *ec*MenB were expressed in *E. coli* BL21 (DE3) with plasmids constructed with the QuickChange Site-Directed Mutagenesis Kit (Stratagene) using the plasmid expressing the wild-type *ec*MenB as the template. The following oligodeoxynucleotide primers were used for the mutagenesis reactions: CCGGTGGTGAGGCAGTGCGTGGTGATTACG and CGTAATCACCACGCACTGC CTGGTCACCACCGG for K89A; GAAGGTCAGGAA GGTGCCAACGCCTTCAAC CAG and CTGGTT GAAGGCGTTGGCACCTTCCTG ACCTTC for R267A, GAAGGTCGCAACGCCGCCAACCAGAAACGTCAG and CTGACGTTTCTGGTT GGCGGCGTTGCGACCTTC for F270A, and CAA CGCCTTCAACCAGGCACGTCAG CCTGACTT and AAGTCAGGCTGACGTGCCTGG TTGAAGGCGTTG for K273A. The mutant genes were verified not to contain unwanted mutations by full-length DNA sequencing. Similar to the wild-type *ec*MenB [18], the mutant enzymes without any tagging sequences were purified using a combination of ammonium sulfate precipitation, ion-exchange chromatography and size exclusion chromatography using a Sephacryl S-100 column. The purified wild-type *ec*MenB and its mutants were stored at −20°C in 25 mM Tris buffer (pH 8.0) containing 10% glycerol, whereas *sc*MenB was stored at −20°C in 20 mM glycine buffer (pH 9.75) containing 1% glycerol before use for activity assay or crystallization. The *ec*MenB mutants were >95% pure on SDS-PAGE.

### Enzyme activity assay

A previously reported coupled assay was used to determine the DHNA-CoA synthase activity of *sc*MenB, *ec*MenB, and the site-directed mutants in 200 mM phosphate buffer (pH 7.0) in the presence of 20 mM NaHCO_3_ [18], [19]. In the assay, the substrate *o*-succinylbenzoyl-CoA (OSB-CoA) was synthesized *in situ* via addition of MenC and MenE to a reaction mixture containing 3–60 μM of SHCHC, 200 μM ATP, 200 μM CoA-SH, 2 mM DTT and 10 mM MgCl_2_ and incubation at room temperature for 10 min. *ec*MenB or its mutant was then added to the mixture for measuring the DHNA-CoA synthase activity by UV-Vis spectrometer at 392 nm corresponding to the absorption maxima of DHNA-CoA.

### Crystallization, data collection and structure determination and analysis

Crystallization trials were carried out at 294 K using the hanging drop method and a range of commercial screens (Hampton Research). For co-crystallization of *ec*MenB and HNA-CoA, two different shapes of crystals (tetragonal and rod-like) were observed under different crystallization conditions in the screening. The tetragonal crystals diffracted poorly on an in-house Rigaku RAXIS IV++ diffractometer and were therefore not further optimized. Large rod-like crystals with the longest dimension >0.3 mm were obtained in a 1∶1 mixture of a solution containing 200 mM (NH_4_)_2_SO_4_ and 23% PEG 3,350 in 100 mM Bis-Tris buffer (pH 5.5) and a protein solution containing 10% glycerol, 10 mg/ml *ec*MenB, 10 mM NaHCO_3_, and 10 mM HNA-CoA in 25 mM Tris buffer (pH 8.0). The crystals appeared within one week and diffracted to ∼ 3 Å. Crystals with satisfactory diffraction quality were finally obtained by including 20 mM KCl in the protein solution after optimization with the Additive Screen (Hampton Research). Harvested crystals were soaked in a cryo-protectant solution containing the mother liquor plus 20% glycerol and then cryo-cooled in liquid nitrogen. For co-crystallization with *sc*MenB, the HNA-CoA and SA-CoA ligands at a concentration of 5 mM were incubated for 1 h at room temperature with the protein at a concentration of 5 mg/ml and 10 mg/ml, respectively, in 20 mM glycine buffer (pH 9.75) containing 1% glycerol and 10 mM NaHCO_3_ before mixing with precipitant solutions for screening and optimization of crystallization conditions. Single crystals of the protein in complex with HNA-CoA were obtained in a 1∶1 mixture of the protein solution and a solution containing 0.15 M ammonium acetate, 0.3 M ammonium sulfate, and 16% PEG 3350 in 100 mM Bis-Tris buffer (pH 5.70), which was supplemented with 10 mM proline or 10 mM taurine as an additive. Single crystals of the protein in complex with SA-CoA were obtained in a 1∶1 mixture of the protein solution with a solution containing 0.15 M ammonium acetate, 4% Tacsimate (pH 6.0), and 15% PEG 3350 in 100 mM Bis-Tris buffer (pH 6.0). All the *sc*MenB crystals were flash-frozen in liquid nitrogen in the mother liquor containing 20% ethylene glycol as the cryoprotectant.

X-ray diffraction data of the single crystals were collected at the beamline BL17U of the Shanghai Synchrotron Radiation Facility (SSRF). Diffraction images were indexed, integrated, and scaled using HKL2000 [38]. The structures were solved by Molecular Replacement with the program Phaser [39] using previously solved MenB structures (Protein Data Bank (PDB) entry 4ELX for *ec*MenB and 4EML for *sc*MenB) as search models. The models were extended by several rounds of manual model fitting and rebuilding with the program COOT [40] and refined using PHENIX Refinement [41] and REFMAC5 [42]. Noncrystallographic restraints were applied for one round of refinement. Restraints of the ligands HNA-CoA and SA-CoA were generated and optimized for the structure refinement using eLBOW [43]. The overall quality of the structural models was assessed by MolProbity [44] and PROCHECK [45]. All graphics were generated using PyMol [46]. Protein interfaces, surfaces and assemblies service PISA at European Bioinformatics Institute was used for analysis of interactions at protein interfaces [47], [48].

## Results

### Crystallization and structural determination of the MenB-inhibitor complexes

The MenB turnover product DHNA-CoA was previously attempted for incorporation into the protein crystals either through soaking or co-crystallization. These efforts resulted in complexes with the coenzyme A moiety in the substrate binding tunnel, but without electron densities for the naphthenoid component [11], [49]. Considering the susceptibility of the 1, 4-naphthenoid ring of the product to oxidation [27], DHNA-CoA was not used in our co-crystallization experiments. Instead, we used the DHNA-CoA analogs that bind and inhibit *ec*MenB in the same mode as the product inhibitor, such as 1-hydroxy-2-napthoyl-CoA (HNA-CoA) and salicyloyl-CoA (SA-CoA) [27]. *ec*MenB was found to readily co-crystallize with HNA-CoA, resulting in large rod-like single crystals in pale yellow. *sc*MenB was also found to form single crystals in the presence of either HNA-CoA or SA-CoA. The *ec*MenB:HNA-CoA complex structure was solved by molecular replacement and refined with PHENIX to a resolution of 1.84 Å, while the structures of the yellowish *sc*MenB: HNA-CoA and colorless *sc*MenB: SA-CoA crystals were also solved by molecular replacement and refined with REFMAC5 to a resolution of 2.35 Å and 2.00 Å, respectively. Data collection and final refinement statistics are summarized in Table 1.

**Table 1:** Data collection and refinement statistics.^a^

	*ec*MenB: HNA-CoA	*sc*MenB: HNA-CoA	*sc*MenB: SA-CoA
PDB ID	4I42	4I52	4I4Z
Data Collection			
Space Group	*P*2_1_2_1_2_1_	*P*3_2_21	*P*3_2_21
Unit Cell Dimensions			
a, b, c (Å)	82.6, 141.6, 288.5	138.8, 138.8, 221.2	139.2, 139.2, 221.0
α, β, γ (°)	90, 90, 90	90, 90, 120	90, 90, 120
Redundancy	5.0 (4.7)	9.1 (8.9)	7.3 (7.2)
Completeness (%)	99.6 (99.5)	99.7 (99.6)	99.6 (99.0)
Reflections (unique)	1360235 (286614)	935568 (102666)	1220915 (166884)
I/σ_I_	10.5 (2.3)	14.3 (3.0)	12.8 (2.9)
R_merge_	0.14 (0.83)	0.14 (0.83)	0.13 (0.63)
Refinement			
Resolution Range (Å)	44.8–1.84	46.6–2.35	44.6–2.00
No. of Atoms	30129	19971	20593
Protein	26560	18830	19136
Water	2476	583	890
Ligands/ions	1101	558	567
Average B Factor (Å^2^)	26.3	43.1	27.9
Protein	26.0	43.1	26.9
Water	36.4	42.2	37.3
Ligand	25.5	44.7	46.7
* R*_work_/*R*_free_	0.15/0.18	0.23/0.26	0.16/0.21
Deviation from ideal values in			
Bond Length (Å)	0.007	0.006	0.007
Bond Angle (^o^)	1.03	1.30	1.09
Ramachandran statistics^b^	98.1%/1.9%/0%	98.5%/1.5%/0%	98.0%/2.0%/0%

a Values for the highest resolution shell are given in parentheses.

b Ramachandran statistics indicate the fraction of residues in the most favored, allowed, and disallowed regions of the Ramachandran diagram.

### Overall structure

**Figure 2:**
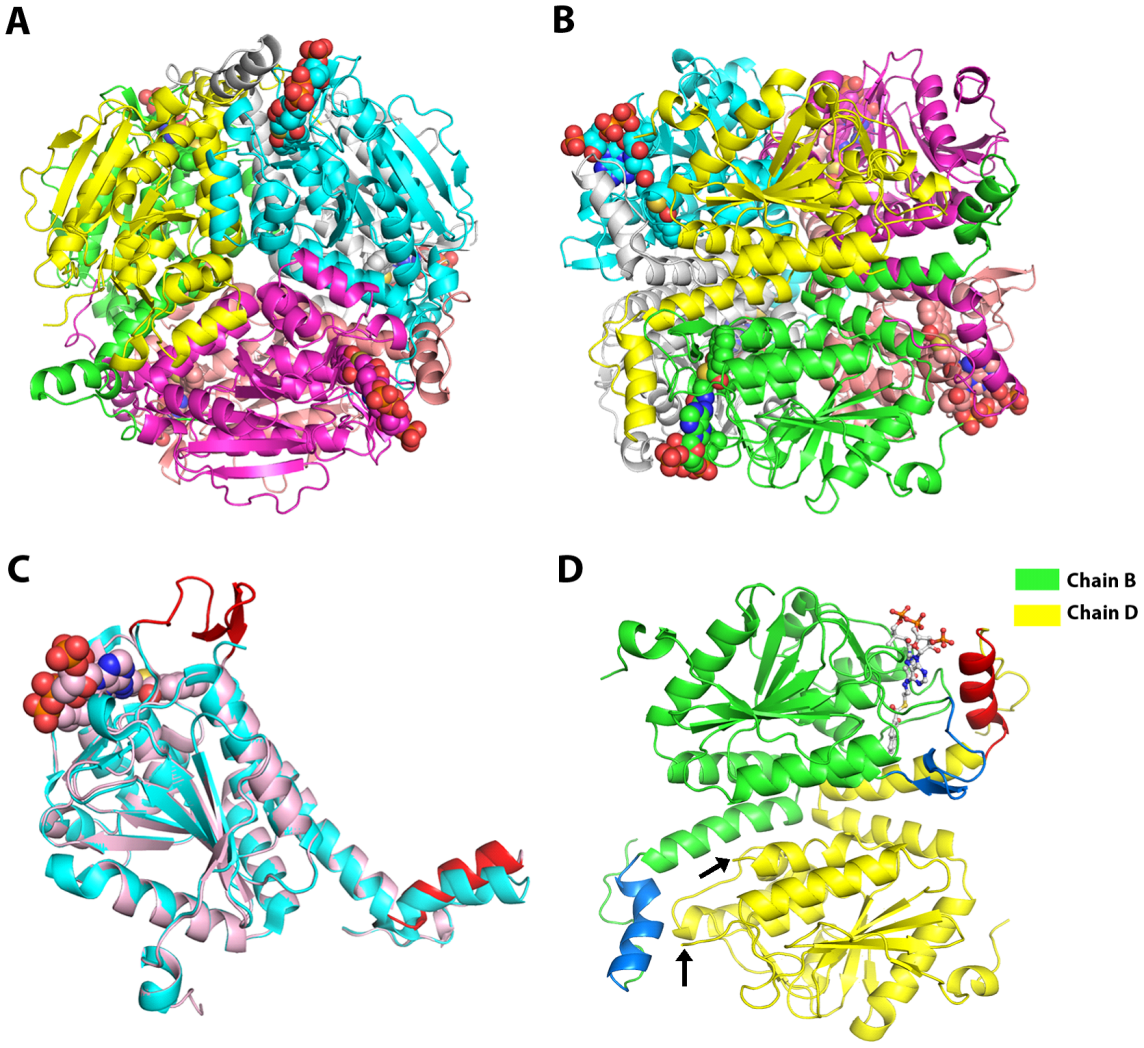
Overall structure of the *ec*MenB: HNA-CoA complex. (A) The hexameric assembly of *ec*MenB:HNA-COA complex viewed along the 3-fold axis of the trimers. The protein is colored by chain and HNA-CoA is shown in sphere. (B) Side view of the hexamer in (A) along the trimer-trimer interface. (C) Superposition of a typical subunit of *ec*MenB: HNA-CoA (in salmon and red with the ligand in spheres) and a subunit of *apo*-ecMenB (PDB code: 4ELX, in cyan). The ordered A-loop (residues 88–105) and the re-oriented C-helix (residues 260–272) are highlighted in red. (D) Partial conformational changes in a ligand-binding subunit (chain B in green and marine) and its opposing unliganded subunit (chain D in yellow and red) across the trimer-trimer interface. Chain B exhibits an ordered A-loop (in marine) and an unchanged C-helix (in marine), whereas chain D contains a disordered A-loop (the two ends are indicated by arrows) and a reoriented C-helix (in red). The HNA-CoA ligand in chain B is shown in sticks.

There are two hexamers in an asymmetric unit of the *ec*MenB: HNA-CoA complex structure with their three-fold axes tilted with each other at an angle of <5°, similar to the arrangement of two hexamers in the asymmetric unit of the crystal of *Mycobacterium tuberculosis* MenB in complex with acetoacetyl-CoA [11]. These hexamers are both composed of two trimers in an eclipsed arrangement and have almost identical quaternary structures (Figures 2A and 2B) with root mean square deviations (rmsd) of 0.17 Å over all Cα atoms. Each subunit of the hexamers consists of an *N*-terminal spiral core domain (residues 1–225) and three *C*-terminal helices (residues 225–272) that extend across the trimer-trimer interface to form part of the active site of another subunit in the opposing trimer, consistent with the general structural feature of all known MenB crystal structures.

The two hexamers in the asymmentric unit of the *ec*MenB: HNA-CoA are different in binding of the small molecule ligand. All subunits in one hexamer are saturated with the ligand in 1∶1 ratio and overlap very well with each other with a rmsd <0.18 Å over all Cα atoms, whereas only five subunits in the other hexamer bind HNA-CoA and no electron density of the ligand is found in the sixth subunit (chain D). When the subunits in the first hexamer are superimposed to those in the *apo*-*ec*MenB structure (PDB code: 4ELX) as shown in Figure 2C, it is obvious that binding of the small molecule ligand causes two large-scale conformational changes in the protein structure. One change is that a disordered active site loop (residues 88–106), called A-loop, in the unliganded protein is folded into a β-turn followed by a β-hairpin when the ligand is bound. The other structural change is a significant reorientation of the C-terminal helix (called C-helix, residues 260–272) of the opposing subunit by an angle of ∼15° towards the enzyme active site. These structural effects of the ligand binding are best demonstrated by comparing the structure of the unliganded subunit (chain D) with its opposing, liganded subunit (chain B) across the trimer-trimer interface in the second hexamer. As shown in Figure 2D, the unliganded subunit has a disordered A-loop and its C-helix is bent towards the active site of the opposing subunit with the small molecule ligand. In contrast, the liganded subunit contains the ordered A-loop and its C-helix is not bent towards the empty active site of the unliganded subunit. These structural differences between the two opposing subunits provide unambiguous evidence that the observed conformational changes are induced by binding of the small molecule ligand.

Both *sc*MenB: HNA-CoA and *sc*MenB: SA-CoA crystals contain nine protein subunits in an asymmetric unit, which are organized into three separate trimers in the former complex and one hexamer and one separate trimer in the latter complex. Through symmetry operation, all the trimers are found to be a part of the hexameric quaternary structure of *sc*MenB. Each subunit in these structures is bound to a small molecule ligand and is essentially identical in conformation with an rmsd <0.20 Å over all Cα atoms. *sc*MenB protein in complex with either HNA-CoA or SA-CoA undergoes the same ordering of the A-loop and the same reorientation of the C-helix as found in the *ec*MenB: HNA-CoA complex structure, when compared with *apo*-*sc*MenB (PDB code: 4EML).

### Active site

**Figure 3:**
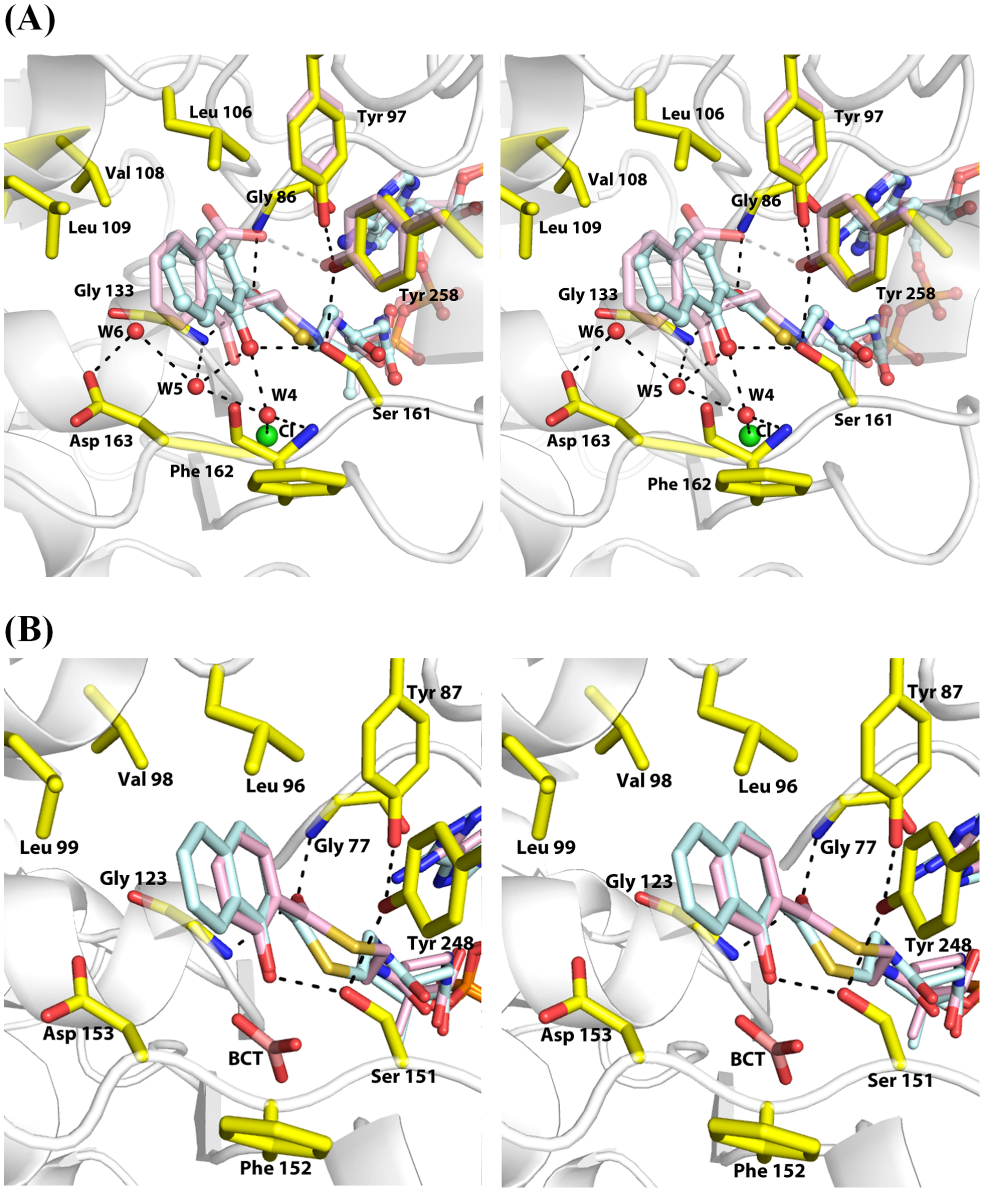
Interactions between the acyl moiety of the ligands and active site residues in *ec*MenB (A) and *sc*MenB (B) complexes. Side chains of the conserved active site residues are shown in sticks with carbon atom colored yellow. Water molecules are shown in red spheres and chloride anion is shown in green sphere. Dashed lines indicate potential hydrogen bonds with a distance less than 3.5 Å. In (A), the protein structure of *ec*MenB: HNA-CoA is represented in gray cartoon and HNA-CoA is represented as sticks with C, O, N, S and P atoms colored light blue, red, blue, brown and orange, respectively. The substrate analog OSB-NCoA and the side chains of Tyr-258 and Tyr-97 from the *ec*MenB: OSB-NCoA structure (PDB code: 3T88) are superimposed onto the *ec*MenB:HNA-CoA structure and shown with all carbon atoms colored in pink. In (B), the protein structure of *sc*MenB: SA-CoA is represented in gray cartoon and the ligands SA-CoA and bicarbonate are represented as sticks with C, O, N, S and P atoms colored pink, red, blue, brown and orange, respectively. HNA-CoA from the *sc*MenB:HNA-CoA structure is superimposed onto the *sc*MenB:SA-CoA structure and shown with all carbon atoms colored in light blue.

The conserved active-site residues in *ec*MenB: HNA-CoA take the same positions and orientations as those in the previously reported *ec*MenB: OSB-NCoA structure [15], despite the obvious structural difference in the small molecule ligands. These residues include Gly-86 and Gly-133 that form an oxyanion hole and are hydrogen-bonded to the thioester carbonyl oxygen of the HNA-CoA ligand; Leu-106, Val-108, and Leu-109 with their hydrophobic side chains interacting with the naphthalene ring of the ligand; and Ser-161 of which the side-chain hydroxyl group forms a hydrogen bond with C1-OH of HNA-CoA (Figure 3A). Another similarity between the two *ec*MenB complexes is the binding of chloride ions at the previously identified bicarbonate-binding sites [16], [18] in both structures. Moreover, there are similar hydrogen bonding networks involving the side-chain carboxyl group of the catalytically essential Asp-163 residue in both complex structures. In *ec*MenB: HNA-CoA, this hydrogen bond network includes the backbone carbonyl oxygen of Phe-162, the backbone amide of Gly-133, three water molecules, and the C1-OH of the HNA-CoA ligand in addition to the Asp-163 side chain (Figure 3A).

However, there are differences between the active sites of the *ec*MenB: HNA-CoA and *ec*MenB: OSB-NCoA complexes. One difference is that the aromatic rings of the two ligand molecules are tilted with each other at an angle of ∼15°, which may affect the interactions of the ligand with the conserved hydrophobic patches consisting of Leu-106, Val-108, and Leu-109. Another major difference lies in the interactions between the ligands and an active-site motif, the latter of which consists of the conserved Tyr-97 from the ordered A-loop and Tyr-258 from the opposing subunit in the *ec*MenB: OSB-NCoA complex. The position and orientation of these two tyrosine residues show little difference in these two enzyme-inhibitor complexes (Figure 3A). Nevertheless, the phenolic hydroxyl groups of these two residues form a hydrogen bond with an O−H⋅⋅⋅O distance of 3.0 Å without interacting with the ligand in the *ec*MenB: HNA-CoA complex, but form two short strong hydrogen bonds with the *ortho*-carboxyl group of the OSB-NCoA ligand in the *ec*MenB: OSB-NCoA complex [15].

The active site of *sc*MenB in complex with HNA-CoA is closely similar to that of *ec*MenB in complex with HNA-CoA. The similarities include the hydrogen-bonding stabilization of the carbonyl oxygen in the ligand by backbone amides of Gly-77 and Gly-123, hydrophobic interactions between the aromatic ring in the ligand with side chains of Leu-96, Val-98, Leu-99, and the hydrogen bonding interaction between the phenolic hydroxyl of the ligand and the side-chain hydroxyl of Ser-151 (Figure 3B). In addition, there is also a hydrogen-bonding network interacting with C1-OH of HNA-CoA, which involves the carboxyl side chain of Asp-153, the backbone amide of Gly-123, three water molecules and the backbone carbonyl oxygen of Phe-152 (data not shown). Moreover, the hydroxyl groups of Tyr-87 from the ordered active-site loop and Tyr-248 from the opposing subunit also form a strong hydrogen bond with an O−H⋅⋅⋅O distance of 2.9 Å. Furthermore, in the *sc*MenB: HNA-CoA structure a chloride ion occupies the bicarbonate binding site (data not shown), similar to that in the *ec*MenB:HNA-CoA complex.

The active site of *sc*MenB in complex with SA-CoA is also very similar to that of the *ec*MenB:HNA-CoA and *sc*MenB:HNA-CoA complexes. However, no hydrogen-bonding network is found in the *sc*MenB:SA-CoA structure. In addition, a bicarbonate ion is found in the place of the chloride ion in the *ec*MenB:HNA-CoA or *sc*MenB:HNA-CoA structure. From these differences, it appears that the hydrogen-bonding networks found in the HNA-CoA and OSB-NCoA structures are related to the presence of chloride ions at the bicarbonate binding sites. Since chloride is not the cognate ligand, the found hydrogen bonding networks are unlikely involved in catalytic mechanism of the enzymes. From the observed crystal structures, it is not clear why the MenB enzymes favor a chloride at the bicarbonate binding site when they bind HNA-CoA or OSB-NCoA.

### Ligand-induced loop-helix interactions

**Figure 4:**
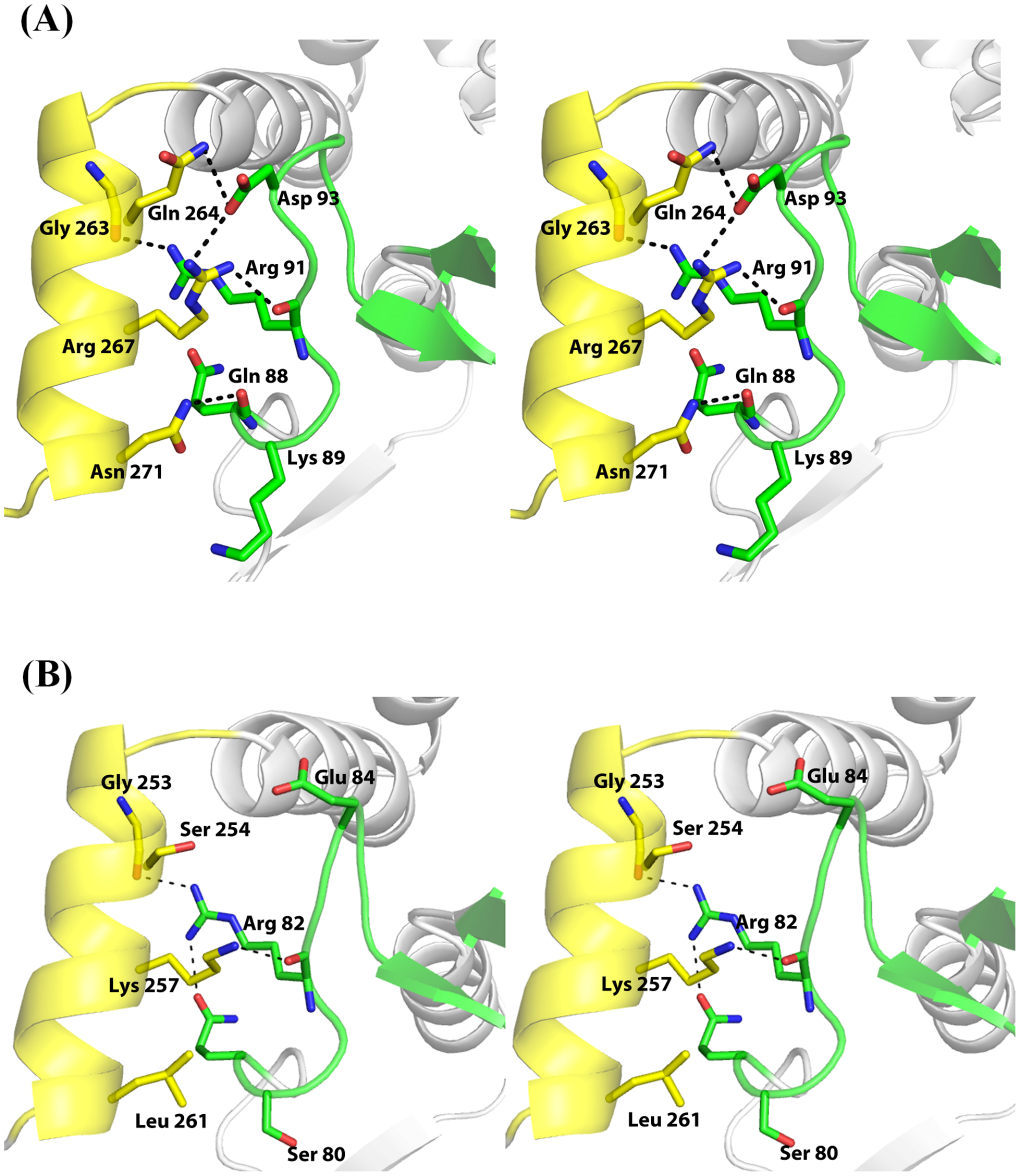
The interactions between the ordered A-loop and the reoriented C-helix in *ec*MenB:HNA-CoA (A) and *sc*MenB:HNA-CoA (B). Residues located in the loop-helix interface are displayed in sticks with carbon atoms colored yellow for the A-loop and green for the C-helix. Dashed lines represent distances less than 3.5 Å.

A close examination of the ordered A-loop and the reoriented C-helix of the opposing subunit revealed that these two structural segments come into direct contact with each other in the enzyme-inhibitor complexes. This ligand-induced loop-helix interaction generates a new interface of 257 and 187 Å^2^ in the inhibitor complexes of *ec*MenB and *sc*MenB, respectively. Analysis of these new interfaces with PISA leads to identification of strong polar interactions involving a strictly conserved arginine residue, which is located in the middle of the ordered A-loop. In the *ec*MenB: HNA-CoA structure, the guanidinium side chain of this residue, Arg-91, forms a strong hydrogen bond (2.9 Å) with the backbone carbonyl oxygen of Gly-263, which is located at the hinge region of the reoriented C-helix. On one side of this hydrogen bond are a salt bridge formed by side chains of Asp-93 and Arg-267 and a hydrogen bond between the side chains of Gln-264 and Asp-93. On the other side of the Arg-91/Gly-263 hydrogen bond are another two hydrogen bonds: one is formed between the backbone carbonyl oxygen of Arg-91 and the side chain of the C-helix Arg-267 and the other is between the side chain amide of the C-helix Asn-271 and the backbone carbonyl oxygen of the A-loop Gln-88 (Figure 4A).

In comparison, there are only two polar interactions in the loop-helix interface of the inhibitor complexes of *sc*MenB. One of them is a strong hydrogen bond between the strictly conserved A-loop Arg-82 and the backbone carbonyl oxygen of the C-helix Gly-253, and the other polar loop-helix interaction is a hydrogen bond between the backbone carbonyl oxygen of Arg-82 and the side chain guanidinium group of Lys-257 (Figure 4B). The decrease in polar contacts at the *sc*MenB interface is at least partially compensated by increased hydrophobic interactions. For example, the side chain of *sc*MenB Leu-261 on the C-helix forms hydrophobic contact with the A-loop, which replaces the interfacial hydrogen bond formed by the corresponding amino acid residue Asn-271 on the *ec*MenB C-helix.

### Additional enzyme-ligand interactions

**Figure 5:**
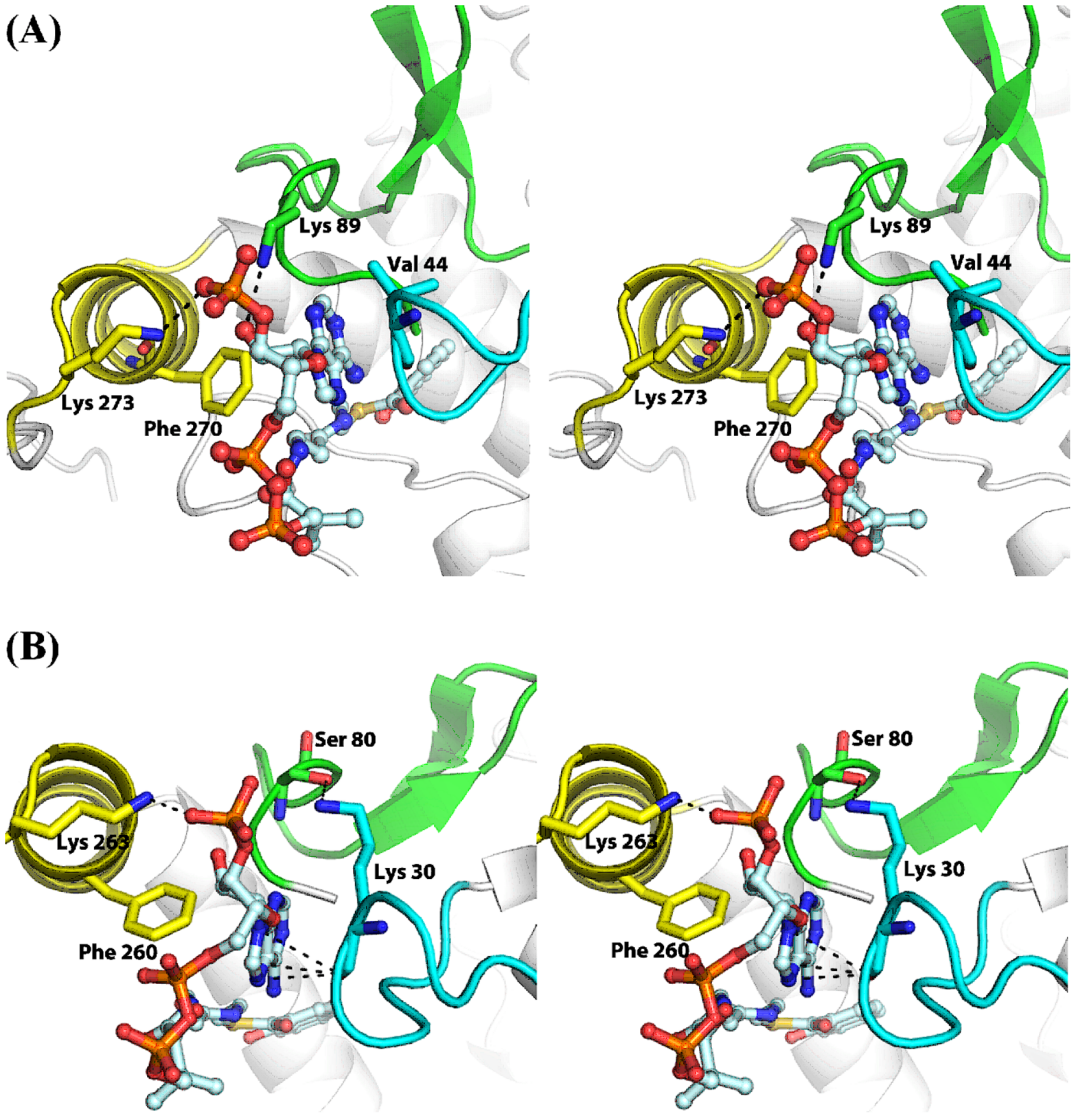
New interactions between the coenzyme A thioesters and the ligand-induced loop-helix assembly in the *ec*MenB:HNA-CoA (A) and *sc*MenB:SA-CoA (B) complexes. The ordered A-loop is colored green and the C-helix is colored yellow. The loop connecting the second β-sheet and the first α-helix at the *N*-terminus, called loop 2, is colored blue. The ligands are represented as sticks with C, O, N, S and P atoms colored light blue, red, blue, brown and orange, respectively. Amino acid residues involved in interaction with the ligands are in stick representation with carbon atoms in the same color as the substructures. Dashed lines represent distances less than 3.5 Å.

As a result of the ligand-induced loop-helix interactions, additional polar and nonpolar interactions are also found at the enzyme ligand interface. In the *ec*MenB: HNA-CoA structure, the side-chain of Lys-89 of the ordered A-loop is found to form a hydrogen bond with the 2′-OH of the ribose ring of the ligand (Figure 5A). In addition, the ligand-induced reorientation of the C-helix results in several additional contacts with the ligands. The first is a salt bridge between the Lys-273 side chain and the adenylate 3′-phosphate of the ligand. The second is a water-mediated hydrogen bond between Lys-273 and the 2′-OH of the adenylate ribose ring in the ligand. Moreover, some hydrophobic contacts are formed between the benzene ring of the Phe-270 side chain and the adenine ring of the ligand, which are almost perpendicular to each other. Obviously, these additional interactions increase the enzyme-ligand affinity. Interestingly, similar salt bridge and hydrophobic interaction are found in the binding of CoA ligands to cause conformational changes in both human mitochondrial monofunctional Δ^3^-Δ^2^-enoyl-CoA isomerase [50] and the crotonase domain of the rat peroxisomal multifunctional enzyme type I [51].

*sc*MenB forms identical polar and nonpolar contacts with the adenylate moiety of HNA-CoA and SA-CoA in the complexes, but shows differences when compared to *ec*MenB. In the *sc*MenB complexes, the side chain benzene ring of Phe-260, which corresponds to *ec*MenB Phe-270, also forms hydrophobic contact with the adenine ring of the ligands. In addition, the adenylate 3′-phosphate group of the ligand is also stabilized by a ligand-induced salt bridge with the side chain of *sc*MenB Lys-263, which is equivalent to *ec*MenB Lys-273 (Figure 5B). However, there is no hydrogen bond like that involving the Lys-89 side chain in the *ec*MenB: HNA-CoA structure. The equivalent residue of *ec*MenB Lys-89 is a serine (Ser-80) in *sc*MenB. Interestingly, the Ser-80 side-chain hydroxyl forms a hydrogen bond with the positive side chain of Lys-30 in the *sc*MenB complexes, which is located in the middle of a 10-residue loop (named loop 2) connecting the second β-sheet and the first α-helix at the *N*-terminus. Due to this hydrogen-bonding interaction, the carbonyl oxygen of Lys-30 is within a short distance of 3.2−3.6 Å from the atoms of the five-membered heterocycle of adenine moiety of the ligand, forming a lone pair (lp)−π interaction that is commonly found in small molecules, nucleic acids and proteins [52]–[54]. In contrast, Val-44 takes the equivalent position of *sc*MenB Lys-30 in the *ec*MenB: HNA-CoA complex, and its carbonyl oxygen is unable to form a similar lp−π interaction by being >3.5 Å away from the adenine ring (Figure 5A).

### Site-directed mutagenesis

To assess the contribution of the observed conformational changes to catalysis of the enzymes, we chose the *ec*MenB residues crucial to the ligand-induced structural changes for site-directed mutation into alanine. These residues include Arg-91 and Arg-267 at the helix-loop interface and Lys-273, Phe-270, and Lys-89 that forms additional interactions with the adenylate moiety of the HNA-CoA ligand. The K273A mutant formed inclusion bodies and was not available for kinetic characterization. All other mutants were readily obtained in a stable and pure form with a conformation similar to the wild type protein as indicated by circular dichroism spectroscopy.

The R91A mutant was found to be inactive at a concentration up to 400 µM, consistent with a previous report [27]. As shown in Table 2, all other mutants are active with a significantly decreased activity as a DHNA-CoA synthase. In comparison with the wild-type enzyme, these mutants exhibit a 5.9−15 fold increase in *K_M_* and a decrease of the catalytic efficiency by 8.3−45 fold. The large *K_M_* increase is indicative of a significantly decreased affinity of the mutants for the substrate. These results show that the amino acid residues crucial to the observed ligand-induced conformational changes are also critical to the catalytic activity of the enzyme, implicating that the ligand-induced conformational changes, or induced fit, is an essential part of the catalytic mechanism.

**Table 2:** Kinetic constants of the *ec*MenB mutants.

Protein	*K*_M_ (µM)	*k*_cat_(min^−1^)	*k*_cat_/*K*_M_ (M^−1^⋅s^−1^)	Relative *k*_cat_/*K*_M_
Wild type^a^	2.8±0.3	1.24±0.06	(7.4±0.5) ×10^3^	1.0
K91A^b^	nd	nd	nd	0.0
F270A	19.7±3.9	0.21±0.01	(1.8±0.1) ×10^2^	0.024
R267A	16.6±1.0	0.16±0.01	(1.6±0.2) ×10^2^	0.022
K89A	43.3±2.0	2.30±0.08	(8.9±0.3) ×10^2^	0.12

a the data was determined in the presence of 20 mM NaHCO_3_ as reported in Ref. 18.

b nd  =  not detectable; the result is consistent with the report in Ref. 27.

## Discussion

The high-resolution crystallographic structures of the DHNA-CoA synthases obtained here have revealed the conformational changes caused by the binding of the product analog HNA-CoA or SA-CoA. The structural changes include the ordering of the active site A-loop, which was also observed in the structure of *ec*MenB in complex with the substrate analog OSB-NCoA [15], and a significant reorientation of the C-helix. The altered A-loop and C-helix are found to interact strongly with each other and to make additional contacts with the small-molecule ligand to strengthen the binding. Actually, all these structural changes are also present in the *ec*MenB:OSB-NCoA structure (PDB code: 3T88) [15], suggesting that these ligand-induced changes are not specific for the product analog inhibitors but are intrinsic property of the enzymes. These findings have shed light on the structural basis for the induced fit of the DHNA-CoA synthases during their catalysis.

There are several possible mechanisms through which the analogs of the substrate OSB-CoA or the product DHNA-CoA cause the observed large conformational changes. In one potential mechanism, the structural changes could occur sequentially starting from ordering of the A-loop, which is initiated by the binding interactions of the acyl moiety of the small molecule ligands with the enzyme active site. Subsequently, the ordered A-loop could induce reorientation of the C-helix to form a new interface. Finally, the newly configured loop-helix assembly generates new interactions with the coenzyme A moiety of the ligand to strengthen the enzyme-inhibitor interactions. In this possible mechanism, the A-loop ordering cannot be due to the direct hydrogen-bonding interaction between the ligand and the conserved residue equivalent to *ec*MenB Tyr-97 in the loop as suggested by Tonge and coworkers [15], because the product analogs used in this study do not directly interact with the conserved residue but cause exactly the same structural changes. Instead, the A-loop ordering may be caused by subtle structural changes of the enzyme active site as a result of the strong interactions identified for the acyl moiety of the ligands (Figure 3). In this connection, it is particularly worth mentioning that the hydrophobic patch composed of Leu-106, Val-108 and Leu109 in *ec*MenB or Leu-96, Val-98 and Leu-99 in *sc*MenB, is connected to one end of the disordered A-loop. Conceivably, the interaction of this hydrophobic patch with the aromatic ring of the ligand may trigger ordering of the loop, which can be subsequently stabilized by the strong hydrogen bond between the strictly conserved loop residue corresponding to *ec*MenB Tyr-97 and the conserved tyrosine (*ec*MenB Tyr-258) from the opposing subunit.

Alternatively, the sequential conformational changes could occur in a reversed order starting from reorientation of the C-helix, which must be initiated by the enzyme-ligand interactions happening at the coenzyme A moiety of the ligand. However, this possibility is excluded by the absence of the A-loop ordering in the complexes of acetoacetyl-CoA with either *Mycobacterium tuberculosis* MenB (*mtb*MenB) [15] or *Staphylococcus aureus* MenB (*sa*MenB) [12], of which the ligand differs from the substrate or product analogs only in the acyl moiety. The inability of acetoacetyl-CoA to cause the conformational changes strongly suggests that the enzyme-ligand interactions at the acyl part of the small molecule ligand are involved in initiation of the large-scale structural changes.

Besides the sequential mechanisms, the ligand-induced conformational changes could also occur in a concerted manner, in which the enzyme-ligand interactions at both the acyl and coenzyme A moieties act synergistically to effect simultaneous A-loop ordering and C-helix reorientation. Currently, no experimental evidence is available to distinguish this concerted mechanism from the sequential mechanism that uses the acyl moiety to initiate the structural changes.

The same ordering of the A-loop and reorientation of the C-helix have been observed for two MenB orthologues in complex with three different ligands, including HNA-CoA and SA-CoA in this study and OSB-NCoA in a previous investigation [15]. These experimental results suggest that the observed induced fit is a conserved feature of all DHNA-CoA synthases that may play an important role in the catalytic mechanism. However, the structural elements directly involved in the induced fit exhibit a high level of variation. At the ligand-induced loop-helix interface, there are two and four hydrogen bonds in the *sc*MenB and *ec*MenB complexes, respectively, of which only the one involving the strictly conserved residue (*ec*MenB Arg-91 or *sc*MenB Arg-82) is identical. In the *sc*MenB complexes, increased hydrophobic interaction at least partially compensate for the binding energy loss due to the fewer polar interactions, demonstrating the plasticity of the loop-helix interface.

**Figure 6:**
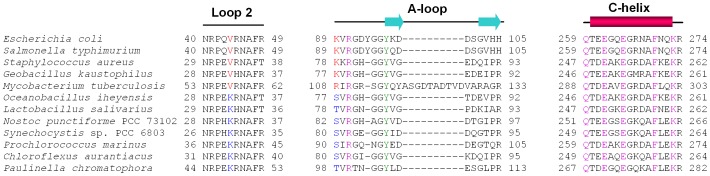
Conservation of the MenB residues involved in the induced loop-helix and protein-ligand interactions. These residues are distributed on the A-loop, C-helix, and Loop 2: the purple residues are conserved among >95% of MenB orthologues and directly involved in the ligand induced interactions; the green residue is a strictly conserved active-site tyrosine; the red residues are conditionally conserved among 113 of the 140 MenB sequences identified in Ref. 18 to form a hydrogen bond between the A-loop and the ribose ring of the ligand as observed for *ec*MenB (Figure 5A); and the blue residues are conditionally conserved among 23 of the 140 MenB sequences identified in Ref. 18 to form an lp−π interaction as observed for *sc*MenB (Figure 5B). The presented sequences are aligned using Clustal W [55]. The secondary structures above the sequences are from the *ec*MenB:HNA-CoA structure, which are represented in arrows for β-pleated sheets, in rectangles for α-helices, and in lines for random coils.

Similar structural plasticity is found for the interface between the coenzyme A moiety of the ligand and the newly configured loop-helix assembly. At this interface, there are two identical direct contacts involving the strictly conserved residues at the equivalent positions of *ec*MenB Phe-270 and Lys-273 and an orthologue-specific polar interaction. In the *sc*MenB complexes, the lp−π interaction is formed between the purine moiety of the ligand and the backbone carbonyl oxygen of Lys-30 accompanying the hydrogen bond between the Lys-30 side chain and Ser-80 (Figure 5B). In contrast, a hydrogen bond is formed between the ribose moiety of the ligand and the side chain of Lys-89 in the *ec*MenB:HNA-CoA complex, which occupies the equivalent position of *sc*MenB Ser-80. No lp−π interaction is formed between the ligand and the backbone of Val-44, which is at the equivalent position of Lys-30 in *sc*MenB. Interestingly, these two distinct interactions appear to be compensatory to maintain a comparable total strength of the interfacial interaction and are conditionally conserved among the 140 MenB orthologues analyzed previously [18]. A lysine and a serine/threonine are conserved at the equivalent positions of *sc*MenB Lys-30 and Ser-80, respectively, in a small subgroup including 2 orthologues from the vitamin K2 biosynthesis of *Lactobacillus* bacteria and 21 orthologues from the vitamin K2 biosynthesis in photosynthetic cyanobacteria, archaea, and eukaryote. However, a lysine or arginine equivalent to *ec*MenB Lys-89 and a valine or isoleucine equivalent to *ec*MenB Val-44 are conserved among 113 of the 140 analyzed MenB orthologues (Figure 6). This conditional conservation of the amino acid residues among different subgroups of MenB orthologues strongly support that the ligand-induced interface is indeed evolutionarily conserved among both type I and type II MenB orthologues. Noticeably, there are still 14 MenB orthologues that have neither lysine nor arginine residue at the equivalent position of *ec*MenB Lys-89 or *sc*MenB Lys-30, suggesting that there may be other mechanisms to maintain the strength of interaction at the ligand-induced interface.

The direct experimental evidence for the involvement of the observed ligand-induced structural changes in enzyme catalysis comes from site-directed mutagenesis. *ec*MenB Arg-91 is a strictly conserved residue forming a strong hydrogen bond with the backbone amide of Gly-263 at the C-helix terminus, which is expected to play a pivotal role in the formation of the helix-loop interface (Figure 4A). Consistent with a previous report [27], its mutation to alanine (Ala) completely eliminates the DHNA-CoA synthase activity. Mutation of another residue involved in the helix-loop interaction, Arg-267, also results in significant activity decrease (Table 2). Both amino acid residues are far from the enzyme active site and are not in direct contact with any part of the substrate or intermediates. Their effects on the enzyme activity can only be exerted via formation of the loop-helix interface, which is impaired or disabled by their mutation. These mutational results demonstrate that the induced-fit, including the observed ligand-induced A-loop ordering and C-helix reorientation, is indeed essential in the catalytic mechanism of the DHNA-CoA synthases.

Additional evidence comes from the mutation of the residues involved in the additional enzyme-inhibitor interactions in *ec*MenB, namely Lys-89 and Phe-270. These point mutations also lead to a significant decrease in catalytic efficiency, which is mainly due to an increase of *K_M_* (Table 2). The lower effect on the enzyme activity compared with R91A is likely due to the fact that each of these residues contributes a small part to the extensive interactions between the enzyme and the thioester ligands and, therefore, their individual mutation leads to less damage in the induced-fit catalytic mechanism. Nevertheless, these mutational results are also unequivocal evidence that the induced fit is an integral and essential component in catalysis of the DHNA-CoA synthases.

The induced fit plays at least two important roles in the catalysis of DHNA-CoA synthases. First of all, it allows formation of a structural motif like an oxyanion hole for orientation of the OSB-CoA substrate and stabilization of the oxyanion intermediate in the intramolecular Claisen condensation, which involves a conserved tyrosine residue in the A-loop [15]. This structural motif may also be involved in stabilization of the anionic intermediates in later tautomerization steps of the enzyme catalysis (Figure 1B). Secondly, as suggested previously [11], the ligand-induced structural changes protect the reactive intermediates from the solvent molecules by sealing the active site off from the bulk solution after binding of the substrate. However, the speculated reorientation of the side chain of the strictly conserved, catalytically essential Asp-163 in *ec*MenB as a result of the ligand-induced conformational changes [27] is not observed in the enzyme-inhibitor complexes. Noticeably, the almost 1∶1 enzyme to inhibitor ratio found in the crystallographic structures is different from the 2∶1 stoichiometric relationship determined in solution [27]. Further investigation is warranted to delineate the effect of this discrepancy on the catalytic role of the induced fit.

In summary, the atomic details of the large scale conformational changes in the induced fit of the DHNA-CoA synthases are fully revealed here by determination of the crystal structures of their complexes with the product analog inhibitors. The critical catalytic role of the induced fit mechanism is experimentally demonstrated by site-directed mutagenesis of the amino acid residues involved in the ligand-induced structural changes. Revelation of the structural details of the induced-fit allows better understanding of the catalytic mechanism of DHNA-CoA synthases, which will facilitate development of new antibiotics targeting the essential enzymes in the vitamin K biosynthesis. In addition, the establishment of the induced fit mechanism for DHNA-CoA synthases may provide incentives to explore whether similar induced fit plays a role in the catalysis of other members of the crotonase superfamily.
